# A Methodological Evaluation of Volumetric Measurement Techniques including Three-Dimensional Imaging in Breast Surgery

**DOI:** 10.1155/2014/573249

**Published:** 2014-01-08

**Authors:** H. Hoeffelin, D. Jacquemin, V. Defaweux, J L. Nizet

**Affiliations:** ^1^Department of Plastic and Maxillofacial Surgery, CHU de Liège, Domaine Universitaire du Sart Tilman, B. 35, 4000 Liège, Belgium; ^2^Institute of Human Anatomy, University of Liège, Domaine Universitaire du Sart Tilman, B. 35, 4000 Liège, Belgium

## Abstract

Breast surgery currently remains very subjective and each intervention depends on the ability and experience of the operator. To date, no objective measurement of this anatomical region can codify surgery. In this light, we wanted to compare and validate a new technique for 3D scanning (LifeViz 3D) and its clinical application. We tested the use of the 3D LifeViz system (Quantificare) to perform volumetric calculations in various settings (in situ in cadaveric dissection, of control prostheses, and in clinical patients) and we compared this system to other techniques (CT scanning and Archimedes' principle) under the same conditions. We were able to identify the benefits (feasibility, safety, portability, and low patient stress) and limitations (underestimation of the in situ volume, subjectivity of contouring, and patient selection) of the LifeViz 3D system, concluding that the results are comparable with other measurement techniques. The prospects of this technology seem promising in numerous applications in clinical practice to limit the subjectivity of breast surgery.

## 1. Introduction

Breast reconstructive and cosmetic surgery nowadays continues to still be a subjective area where each intervention is surgeon dependent. Currently we note the unfortunate absence of measurement tools that allow objective analysis of this anatomical region, a region that is a particularly representative model of the human variability that limits its study (great variety of shapes, sizes, and compositions). Different volumetric methods of breast measurement (anthropometric formula, Grossman-Roudner method, etc.) have been described in the literature using clinical methods [1–3], casts [4], or the Archimedes principle [5, 6].

The use of medical imaging methods such as mammography [7], sonography [8], and magnetic resonance imagine (MRI) [9] has also been described. However, none of those methods have been reported to be superior to the others. More recently a French group [10] studied a method of optical scanning using structured light projection (Inspeck system) allowing them to assess the advantages and limitations of 3D imaging in breast surgery and concluding that such a volumetric calculation is perfectly suited to clinical practice.

Other groups were interested in comparing [11] different methods of volumetry (mammography, the Grossman-Roudner method, anthropometric calculation, the Archimedes principle, and casts) and demonstrated the superiority of imaging, followed by the Archimedes principle.

Lately, innovative studies [12] suggested the basis for a new biochemical imaging technology (nanodiamond imaging) that noninvasively records the distribution in two or three dimensions of biologically labeled nanodiamonds in vivo. Our study aims to evaluate and compare (using CT-scanning and the Archimedes principle) a new technique of 3D scanning acquired via stereo-visual technology (3D LifeViz) and its application to biometrics of the breast in clinical practice in terms of volume calculation and sternal notch to nipple distance.

Our study included several steps.

- *Experimental section*, consisting of 3 components:
  1. comparison of three volumetric techniques on anatomical prostheses with known volumes (control);
  2. comparison of three volumetric techniques on “anatomical models” (dissection and sampling from cadavers);
  3. evaluation of the LifeViz 3D camera by in situ volumetric analysis and on dissections from cadavers.
- *Clinical section*, with in situ patient acquisition during consultation for cosmetic and plastic surgery for the assessment of the benefits and limitations of this 3D scanning technique.

## 2. Materials and Methods

### 2.1. LifeViz 3D System (Quantificare SA)

**Figure 1:**
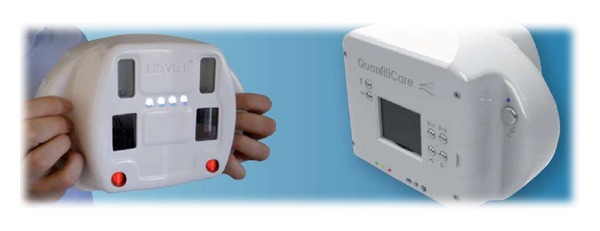
LifeViz 3D Camera.

LifeViz (Quantificare SA, 1180 Route des Dolines, Athena B, BP 40051, 06901 Sophia Antipolis, France) is a system for 3D reconstruction and analysis (Figure 1).

LifeViz is composed of a digital stereo camera, a repositioning device (we used a portable system), and 3D software: LifeViz DermaPix 3D from Quantificare and its quantification platform, LifeViz.

LifeViz has two main features for volume measurements:

1. LifeViz “Absolute” for the evaluation of the volume in a single acquisition;
2. LifeViz “Evolution” for the relative quantification of volume variation.

The principle of LifeViz “Absolute” is the measurement of a cavity or a protrusion using simple image acquisition. It is based on the delineation of the cavity (or protrusion) and then closing the volume with a “minimum surface,” which is the mathematical equivalent of a soap film stretched across the delineated outline.

LifeViz “Evolution” is ideal for evaluation of changes in volume over time (it is well adapted to measure the boundaries of anatomical structures that are difficult to define as in the case of labial region).

### 2.2. Anatomical Specimens

We used nine cadavers of women aged over 60 from the Institute of Human Anatomy (CHU Liège) to measure the volume of each breast in situ and then after dissection (removal of the anatomical section).

We used four “prepared” cadavers that had undergone preparation with zinc chloride and preservatives such as ammonium. These bodies were kept in containers with the above-mentioned products for a period of 12 to 18 months (the cadavers were assigned to the Institute of Human Anatomy for medical student teaching).

Five other cadavers were “fresh” cadavers frozen at −20°C in the cold room without any preparation. We allowed them to thaw at room temperature in the Institute of Human Anatomy (15°C) for 48 hours before acquisition and dissection. These cadavers had been frozen for more than 6 months.

#### 2.2.1. In Situ Volume Calculation from the Cadavers

First we performed a contouring that consisted of delineating the contours of the breast as closely as possible, subluxing the breast along different axes, defining the furrows to be taken as an outline, and then redrawing the contours with indelible ink. The bodies were all in the dorsal decubitus position for easy mobilization, with the upper limbs flexed to minimize the hidden parts of the breast in 3D acquisitions (to minimize the possibility of a measurement bias and therefore a truncated volume).

**Figure 2:**
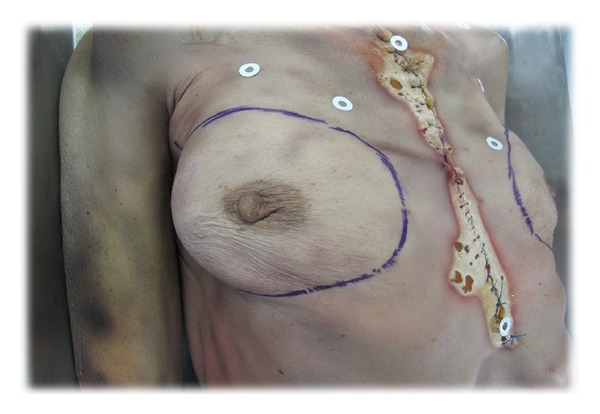
Contouring technique.

We took seven imaging shots in total:

- five views termed “stitching”: front, profile 45° bilaterally, and inferior profile 45° also bilaterally, with a focal distance of 100 cm;
- two views termed the “incident breast” to minimize hidden parts: inferior-lateral profile 30° bilaterally, with the same focal distance as before (Figure 2).

#### 2.2.2. Volume Calculation on Anatomical “Samples” (Experimental)

The breasts were previously marked (left/right) and then carefully resected (as contoured) avoiding taking muscle fibers from the pectoralis major muscle or the serratus anterior muscle. It should be noted that the greatest difficulty of dissection was in the frozen cadavers, where the physiological cleavage planes were almost absent and in which a more difficult but ultimately satisfactory dissection was performed.

The 3D LifeViz acquisition was performed with a single lateral view using the table since images taken perpendicularly could overestimate the density. Anatomical sections were placed on a table covered with textured stainless steel forming the surgical field. It is important to note that the “absolute” measurement with the 3D LifeViz camera is accurate when the closing surface is flat, as is the case in this anatomical resection (i.e., when placed flat on the stainless steel table).

We performed the same procedure for our control measurements on prostheses of known volume (Mentor).

For the volume calculation in situ the closing surface of the 3D volume is somewhat arbitrary.

**Figure 3:**
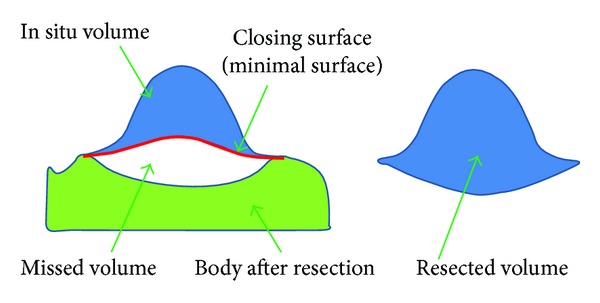
Schematic presentation of in situ underestimation.

**Figure 4:**
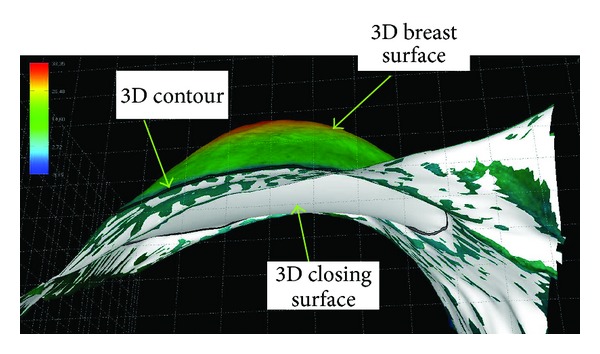
Reconstruction of a breast in situ with 3D LifeViz. Note the shape of the closing surface (white) below. This is a minimum area based on the 3D (black line) contour that is generally concave due to the physiological curvature of the thoracic cage.

**Figure 5:**
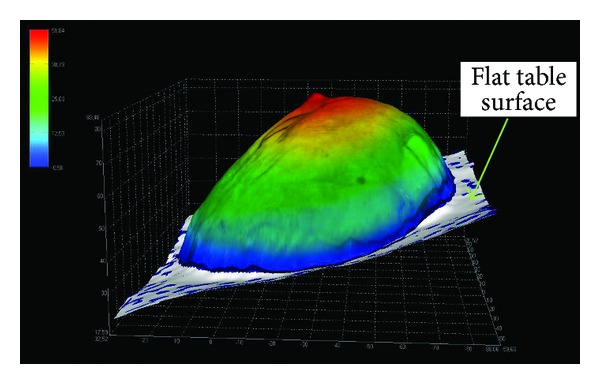
Reconstruction of resected breast with 3D LifeViz (anatomical part). The base is a table surface (map), where the 3D contour is quite flat, as this is also the case for the closing surface (white).

There is a minimum area in a 3D complex shape based on the 3D contour defined by the operator. Due to the physiological curvature of the chest, as well as the fact that resection will create a depression in the body, it is not expected that the in situ measurement will correspond to the measurement of a removed anatomical part (i.e., underestimation of the volume in situ) (Figures 3, 4, and 5).

#### 2.2.3. In Situ Volume Calculation in Patient

We also undertook 3D image acquisition of 38 patients that presented for various breast-related indications at the Department of Aesthetic and Reconstructive Surgery. These patients were seen preoperatively and, if necessary, during irregular postoperative intervals. Patients were informed of the experimental nature of procedures.

The image acquisition procedure comprising seven views was the same as that used in the cadavers, specifically 5 “stitching” views and 2 “incident breast” views.

Patients stood with their back against the wall and with the posterior parts of the shoulders abutting the wall to reveal the chest in its entirety. The elbows were bent and hands were placed on the hips to minimize to the greatest extent any hidden part of the breast. Overall, this position gives a good overview of the region of interest in order to observe both breasts and their proper insertions. The position was not very comfortable for the patient, but the speed of acquisition of less than 1 minute made it tolerable.

**Figure 6:**
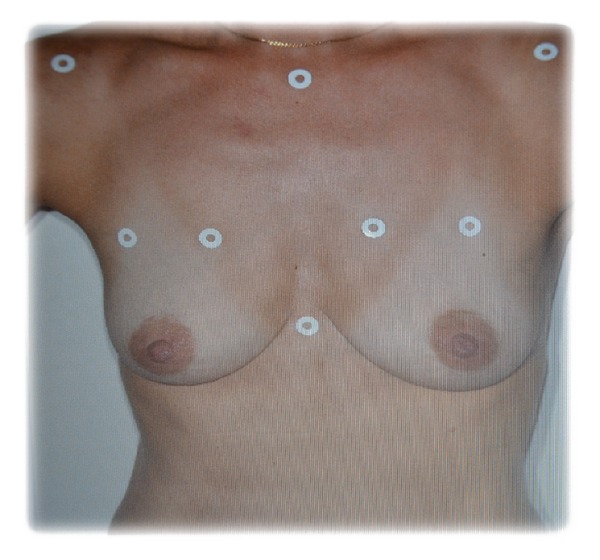
Anatomical spotting technique.

Unable to achieve contouring in our patients during the consultation, we used marking with circular adhesive pads (which were easy to use and safe and had low cost) that were applied in relation to prominent parts of the bones (jugular notch of manubrium sterni (called the “inlet”), the xiphoid appendix, and the acromioclavicular joints bilaterally) and thus delimited the anatomical region of interest (see Figure 6). On the breast itself, the challenge was to define its upper insertion. For this we used a technique of superior subluxation according to Professor P. Blondeel at the University of Ghent (Belgium). The patient raised her breast at its base with the dorsal surface of the ipsilateral hand delimiting an upper portion taken as proximal part of insertion of the breast that we marked with our adhesive pads (Figure 6).

### 2.3. Archimedes' Principle

We used an old method of Archimedes' principle of buoyancy telling; “Any object, when wholly or partially immersed in a fluid, is buoyed by a force equal to the weight of the fluid displaced by the object” (the volume is equal to the immersed volume of the body). This method is attractive because of its simplicity, reproducibility, low cost, and speed of execution. For this we used a Brand measuring cup of 3000 mL/50 mL by graduation. Then each freshly collected anatomical resected breast was immersed in a determined volume of liquid, in this case 2000 mL of water (the volume was the same for all measures). After recording the different displaced fluid values, we satisfactorily calculated the various results. We repeated the maneuver for the 18 breasts (cadaveric) as well as for four anatomic prostheses of known volume.

### 2.4. CT-Scan

For scan acquisitions we used a GE Bright Speed 16 strips, model 2010. For this purpose we used the volumetric software “Paint on Slice,” an application of segmentation program “Advantage Work Station GE 4.6” (basic volume viewer). We undertook volume acquisition in spiral mode (abdomen program) without injection of contrast, in axial sections with cuts of 1.25 mm (thickness) every 1 mm (interval), and with 120 kV for 209 mAs per procedure. The acquisition time was 20 seconds for the first series (anatomical parts) and 13.83 sec for the second series (prostheses).

## 3. Results

### 3.1. Data Handling and Statistics

We measured the volume (cm³) of the left and right breasts of nine cadavers (S1–S9) using our various techniques. The VIZ3D technique used a 3D camera for volume calculation. This technique was performed once in situ (VIZ3D_INSITU) and once on the resected sample (VIZ3D_PREL). The other two techniques were performed only on the resected sample, namely, Archimedes' manual method (ARCHI_PREL) and the CT scan (CTSCAN_PREL).

The volume was also measured by the 3 techniques (VIZ3D_PREL, ARCHI_PREL, and CTSCAN_PREL) in 4 breast implants with different sizes termed controls of known volume (VOLCONNU).

We performed measurements in triplicate on the cadaver or anatomical part, the prosthesis or in vivo, and calculated the mean volume measurement for each method, rounded up to the closest unit. We tested three types of 3D LifeViz cameras that differed only by the focal distance of acquisition of 70, 80, and 100 cm. After testing, we kept the camera focal length of 100 cm to allow for optimal breast measurement in our study.

### 3.2. Data Listing (Table 1)

#### 3.2.1. Methodology

To assess concordance between two series of measurements (technique 1 and technique 2) several statistical methods can be used.

*Coefficient of Intraclass Correlation (ICC). *The ICC is a measure of concordance between two series where the studied variable is continuous (the volume measures in cm³). The closer the ICC is to 1, the better the concordance is between the two series. Zero (ICC = 0) signifies the absence of concordance. 

*Paired Samples Student's t-test/Wilcoxon Signed Rank Test. *The paired samples Student's *t*-test is used to test the hypothesis that the average difference between two measures (techniques) is absent. A *P* value is associated with value obtained from Student's *t-*test. If the test is rejected (*P* < 0.05), that means there is a systematic difference between the values provided by two techniques. Otherwise, we consider that the means in two techniques give the same values. Wilcoxon test of signed ranks is a nonparametric test corresponding to the Student's *t*-test and compares the medians. 

*Reproducibility of Measurements (CV). *To measure the reproducibility of a test, namely, the ability to reproduce the same value by repeating the measurement, we can use two results obtained for each sample as if they had both been obtained using the same technique. Reproducibility is best expressed by a coefficient of variation CV (%). The procedure is as follows: consider *n* pairs of measurements {(*x*_*i*1_, *x*_*i*2_), *i* = 1,…, *N*}. Note that $\overset{-}{x}=({\overset{-}{x}}_{1}+{\overset{-}{x}}_{2})/2$ and *s*² = (∑*d*_*i*_^2^)/(2*n*), where $\overset{-}{x}$ is the mean of means of two measurements and {*d*_*i*_ = *x*_*i*1_ − *x*_*i*2_, *i* = 1,…, *N*}. So, $\text{CV}=(s/\overset{-}{x})\times 100\%$. The lower the CV is, the better the reproducibility is.

*Bland-Altman Plot. *In a Bland-Altman plot, for each pair of measurements {(*x*_*i*1_, *x*_*i*2_),  *i* = 1,…, *N*} we report on the abscissa the mean of two measurements ${\overset{-}{x}}_{i}=\left(x_{i1}+x_{i2}\right)/2$ and on the ordinal the difference *d*_*i*_ = *x*_*i*1_ − *x*_*i*2_. In principle, there should not be any association between *d*_*i*_ and ${\overset{-}{x}}_{i}$. We can test this hypothesis by calculating the correlation coefficient *r* (classical or spearman) between the two series and associate it to the *P* value. The absence of correlation indicates that the gap between two measurements does not vary with volume.

### 3.3. Cadavers: Comparison of VIZ3D In Situ versus VIZ3D Sampling (Table 2)

**Figure 7:**
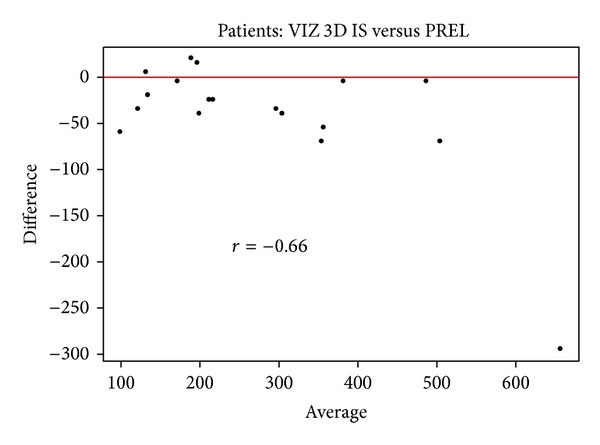
Bland-Altman plot comparing VIZ3D in situ versus VIZ3D sample from the bodies. This graph shows stability of measures; the difference between the measurements does not depend on volume.

There was a significant difference of 41.4 ± 68.9 cm³ (*P* = 0.021) between two methods. The in situ method underestimates the volume compared to the resection-based method. The concordance between the two methods is good (0.87) but the lower confidence limit is not very high. The difference between two methods is 19.9% (Figure 7).

Regarding the underestimation of in situ measurements, additional information could be assessed. The 3D image could be obtained after resection of the anatomical area. This would allow measurement by the 3D LifeViz camera of the hollow anatomical section left by the resection. This would allow one to compare the differences in volume before and after resection, the volume of the resected portion, which would explain the underestimation of the volume due to the hollow left by resection.

For its part, the company trained operator also made measurements of the same areas as us. Note that all their measurements were made with a single view of the 3D camera (instead of 5 + 2 made by us), with the angle that seemed the most appropriate. This was possible in all subjects except one case in situ and in one localization for this patient, where the arm limited this measurement. As a result, the company excluded this particular image of the in situ experiment and therefore used 8 subjects instead of 9 in our series. Their number 7 has only one image on the right side because the left side contained too many hidden regions and therefore could bias the measurement. They did not perform the mean of three measurements (rather, one single measurement in the optimal orientation of imaging was used). Their trained experience in shooting and contouring (one large contour will permit better definition of the closing surface without increasing the measured total volume and has less risk of losing a part of the volume of the object) as compared to “naive” operators is predictable. Their findings after statistical integration are shown. Note that the order of their subjects is not identical to ours (Table 3).

We observed a significant difference of 47.1 ± 36.5 cm³ (*P* = 0.0002) between the two methods. The concordance between the two methods is very good (0.92) but the lower confidence limit is not particularly high. The coefficient of variation between two methods is 14.5%. As expected, the company measurements are more precise and better correlated than ours. This indicated that the measurements are highly operator dependent. Therefore for future clinical use by various practitioners standardized training by the company itself is needed to have reproducibility of measurements (particularly important in clinical follow-up or pre-and postoperative comparison).

### 3.4. Controls: Comparison of Methods (Table 4)

**Figure 8:**
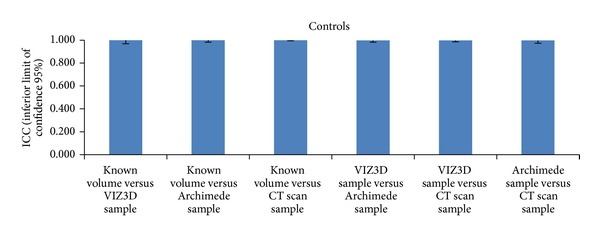
Comparisons of methods (controls) based on ICC values. The results should be assessed with caution because there were only four observations.

We used as “controls” four anatomic Mentor implants with known volume (T1: 60cc, T2: 150cc, T3: 330cc, and T4: 460cc). Using these controls we compared one with another using our three methods of study and we found good concordances and relatively low coefficients of variation. However, the results should be assessed with caution because there were only four observations (Figure 8).

### 3.5. Cadavers: Comparison of Methods Using Resected Samples

In the cadavers (S1–S9), when comparing the volume with the reference method (here it was the CT scan), there was a mean difference of 47.2 ± 39.0 cm³ for VIZ3D method and a mean difference of 13.1 ± 23.6 cm³ for the Archimedes method. In all cases, the difference was statistically significant. The best concordance with the CT scan was obtained with the Archimedes method and with this the ICC was 0.992 and the lower confidence limit was very high (0.978). For the VIZ3D method, we obtained a concordance of 0.951 (0.629) between the samples. For the VIZ3D method, the ICC was high but the lower confidence limit was below that observed for the Archimedes method. The coefficient of variation for the Archimedes method was also lower (5.29%) as compared to the VIZ3D technique (in situ 29.9% and on samples 13.2%).

With these results, we can suggest that imaging is the technique of choice, followed by the Archimedes principle and then the 3D imaging LifeViz.

**Figure 9:**
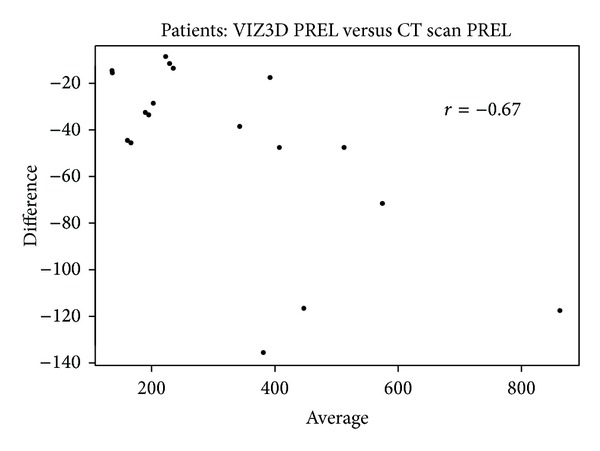
Bland-Altman plot comparing VIZ3D sampling versus CT scan sampling (parts of dissection). When comparing the volume with the reference method (here it was the CT scan), there was a mean difference of 47.2 ± 39.0 cm³ for VIZ3D method.

**Figure 10:**
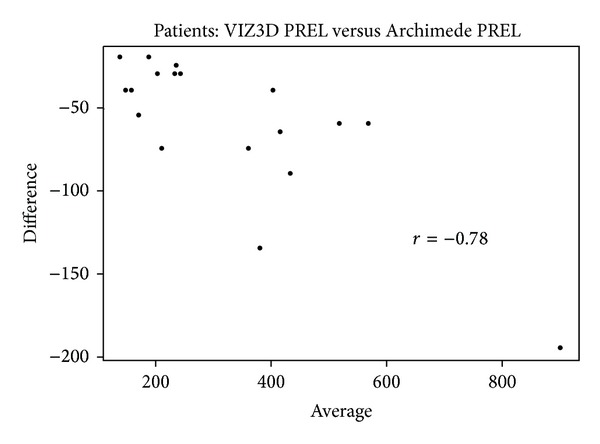
Bland-Altman plot comparing VIZ3D sampling versus Archimedes' method on samples (anatomical dissection). When comparing the volume with the reference method (here it was the CT scan), there was a mean difference of 13.1 ± 23.6 cm³ for the Archimedes method. In all cases, the difference was statistically significant.

**Figure 11:**
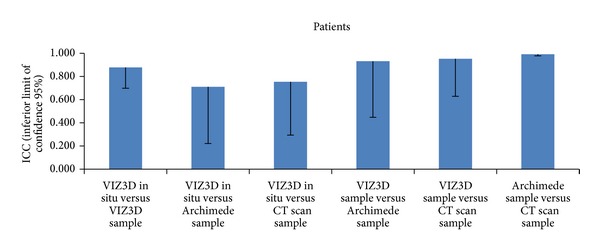
Comparison of methods (cadavers) based on ICC values. The best concordance with the CT scan was obtained with the Archimedes method and with this the ICC was 0.992 and the lower confidence limit was very high (0.978). For the VIZ3D method, we obtained a concordance of 0.951 (0.629) between the samples. For the VIZ3D method, the ICC was high but the lower confidence limit was below that observed for the Archimedes method.

**Figure 12:**
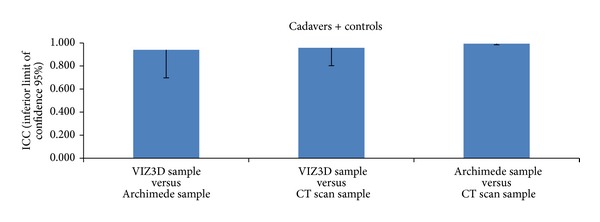
Comparison of methods (cadavers + controls) based on ICC values. If we look at the overall concordance between three methods, we obtain an ICC of 0.96 (inferior limit = 0.90), suggesting comparability of these three methods.

If we look at the overall concordance between three methods, we obtain an ICC of 0.96 (inferior limit = 0.90), suggesting comparability of these three methods (Figures 9, 10, 11, and 12).

### 3.6. Acquisition of Patients' Images

Over a period of a month we scanned 38 patients, preoperatively in the majority of cases. These scans allowed us to identify issues related to the acquisition of patients in clinical practice (during consultation) and to gain the necessary experience with a view to clinical use. Indications for surgery in patients who received at least one 3D acquisition were varied, such as, breast reconstruction by prosthesis and/or lipomodelling, remodeling of breast reconstruction, breast asymmetry, breast ptosis, or breast reduction.

## 4. Discussion

This study evaluates the application of 3D image acquisition to breast surgery using a new technique for 3D stereovision, LifeViz. We demonstrated a good correlation between the measurements made using the CT scan and Archimedes' principle buoyancy with anatomical samples and isolated implants and with the 3D camera. However, the volume calculation in situ (cadaveric and clinical) needs to be improved (due to significant underestimation of volume).

The advantages and limitations of this new technology should be considered.

*Advantages.* (i) Portable device: this is a major advantage of this system that could increase clinical use (the transportation to consultation from one department to another), usage in operating rooms (acquisition could be performed pre-, per, and immediately postoperatively or later), mobility (low weight and acceptable size), and usage in any place (no need of a “special” room). We would emphasize the substantial autonomy for several days of use.

(ii) Safety: no irradiation, a flash similar to those used in photographic cameras and harmless to the human eye, pain-free technique.

(iii) Low stress for the patient: very short acquisition time (of the order of seconds), which increases the quality of measurements by avoiding patient movement (that can produce image distortion). The acquisition can be performed sitting or standing. Here we chose the standing position with hands on hips (intermediate position between the anatomical position and the raised arms position) to minimize the hidden parts of the chest and to maintain the advantages of both positions above (this was possible because our patients were all preoperative); the intermediate position was originally described by Sinna et al. [10].

(iv) Additional value: the advantages over traditional photography (the objective forms angles of view not accessible by standard simple photography).

(v) Cost: <15000 Euros for the entire apparatus.

(vi) Noninvasive technique: noncontact nature of the process (therefore possible to use even on sensitive soft tissue, and it doesn't cause any deformation).

*Disadvantages.* (i) A problem is that the focal length needs to be respected (between 80 and 120 cm), with the risk of image distortion as a penalty (computer contouring difficulties occur in these conditions) and the problem of comparability of results.

(ii) Difficulty in defining breast contours (upper limit is the axillary pillar).

(iii) Underestimation of the volume in situ, hence the difficulty of reliable clinical utilization.

(iv) Selection of patients: patients with significant ptosis or who are significantly overweight disrupt the scans, resulting in the measurement bias by increasing the hidden regions (in particular that of segment III) but also making the breast limits barely perceptible during contouring.

(v) Subjectivity in contouring: contouring is very operator dependent, which makes it difficult to reproduce.

## 5. Conclusion

This technology appears to offer a promising future because of its multiple applications particularly in clinical practice. The technology challenges the subjectivity of surgery, allowing the more likely obtainment of predictable and defined results, improving patient satisfaction and serving as an objective and reliable measurement tool for the practitioner to improve the quality of interventions and outcomes. In our evaluation of the 3D LifeViz camera, in situ volume calculations alone remain perfect for true routine clinical use.

## Figures and Tables

**Table 1:** Data listing.

Group	Name	Side	VIZ3D_INSITU	VIZ3D_PREL	ARCHI_PREL	CTSCAN_PREL	Known vol
Patients	S1	G	205	190	220	219	—
	S1	D	200	180	200	214	—
	S2	L	485	490	550	538	—
	S2	R	380	385	450	433	—
	S3	L	280	315	450	451	—
	S3	R	320	390	480	507	—
	S4	L	70	130	150	146	—
	S4	R	180	220	250	229	—
	S5	L	330	385	425	403	—
	S5	R	285	325	400	364	—
	S6	L	105	140	180	185	—
	S6	R	135	130	170	145	—
	S7	L	125	145	200	191	—
	S7	R	170	175	250	208	—
	S8	L	470	540	600	612	—
	S8	R	510	805	1000	923	—
	S9	L	205	230	260	244	—
	S9	R	200	225	250	237	—

Controls	T1		—	75	55	60	60
	T2		—	157	150	146	150
	T3		—	339	350	335	330
	T4		—	461	450	474	460

**Table:** (a)

Name	Side	VIZ3D_INSITU	VIZ3D_SAMP	dVIZisVISp
S1	G	205	190	15
S1	D	200	180	20
S2	L	485	490	−5
S2	R	380	385	−5
S3	L	280	315	−35
S3	R	320	390	−70
S4	L	70	130	−60
S4	R	180	220	−40
S5	L	330	385	−55
S5	R	285	325	−40
S6	L	105	140	−35
S6	R	135	130	5
S7	L	125	145	−20
S7	R	170	175	−5
S8	L	470	540	−70
S8	R	510	805	−295
S9	L	205	230	−25
S9	R	200	225	−25

	Mean ± SD	258.6 ± 133.5	300.0 ± 178.0	−41.4 ± 68.9
	Median			−30
	P25–P75			−55–−5
	SD (robust)			37.0

**Table:** (b)

Method 1	Method 2	*N*	ICC	Difference	Paired Student's *t*-test	Wilcoxon	CV (%)	Bland-Altman
			(ICC*)		*P* value	*P* value		*r* (*P* value)
VIZ3D in situ	VIZ3D sample	18	0.878 (0.698)	−41.4 ± 68.9	0.021	0.0008	19.9	Pearson: −0.66 (0.0031)
								Spearman: −0.39 (0.10)

There was a significant difference of 41.4 ± 68.9 cm³  (*P* = 0.021) between two methods. The in situ method underestimates the volume compared to the resection-based method.

**Table:** (a)

Subject	Side	Volume_in_situ	Volume_resection	diff
P001	Right	384.5	424.7	−40.2
P001	Left	507.7	520.8	−13.1
P002	Right	362.3	436.8	−74.5
P002	Left	276.0	402.3	−126.3
P003	Right	171.5	234.0	−62.5
P003	Left	69.1	156.7	−87.6
P004	Right	285.8	350.1	−64.3
P004	Left	345.1	398.5	−53.4
P005	Right	153.2	133.2	20.0
P005	Left	108.3	173.0	−64.7
P006	Right	168.9	205.0	−36.1
P006	Left	123.8	165.4	−41.6
P007	Right	525.7	574.9	−49.2
P008	Right	244.7	248.2	−3.5
P008	Left	230.3	240.3	−10.0

	Mean ± SD	264 ± 140	311 ± 142	−47.1 ± 36.5
	Median	245	248	−49.2
	P25–P75	153–362	173–425	−64.7–−13.1
	SD (robust)	155	186	38.2

**Table:** (b)

Method 1	Method 2	*N*	ICC	Difference	Paired Student's *t*-test	Wilcoxon	CV (%)	Bland-Altman
			(ICC*)		*P* value	*P* value		*r* (*P* value)
In situ	Resection	15	0.92 (0.42)	−47.1 ± 36.5	0.0002	0.0004	14.5	Pearson: *r* = −0.06 (*P* = 0.82)
								Spearman: *r* = 0.04 (*P* = 0.89)

We observed a significant difference 47.1 ± 36.5 cm^3^  (*P* = 0.0002) between the two methods. The concordance between the two methods is very good (0.92) but the lower confidence limit is not particularly high.

**Table 4:** Comparison of methods (controls).

Method 1	Method 2	*N*	ICC	Difference	Student's *t*-test	Wilcoxon	CV (%)	Bland-Altman
			(ICC*)		*P* value	*P* value		*r* (*P* value)
T1–T4								
Known volume	VIZ3D samp.	4	0.999 (0.968)	−8.00 ± 5.77	0.070	0.13	2.63	Pearson: 0.84 (0.16)
								Spearman: 0.80 (0.20)
Known volume	Archimedes' samp.	4	0.999 (0.982)	−1.25 ± 13.1	0.86	0.99	3.23	Pearson: −0.10 (0.90)
								Spearman: 0.20 (0.80)
Known volume	CT scan samp.	4	0.999 (0.994)	−3.75 ± 7.76	0.41	0.50	2.16	Pearson: −0.899 (0.10)
								Spearman: −0.80 (0.20)
VIZ3D samp.	Archimedes' samp.	4	0.997 (0.982)	6.75 ± 13.0	0.38	0.50	3.65	Pearson:−0.47 (0.53)
								Spearman: −0.40 (0.60)
VIZ3D samp.	CT scan samp	4	0.998 (0.986)	4.25 ± 12.4	0.54	0.63	3.18	Pearson: −0.956 (0.044)
								Spearman: −1.00 (<0.0001)
Archimedes' samp.	CT scan samp	4	0.997 (0.973)	−2.50 ± 16.5	0.78	0.88	4.06	Pearson: −0.34 (0.66)
								Spearman: −0.20 (0.80)

Using these controls we compared one with another using our three methods of study and we found good concordances and relatively low coefficients of variation.
